# Coexpression network analysis of human candida infection reveals key modules and hub genes responsible for host-pathogen interactions

**DOI:** 10.3389/fgene.2022.917636

**Published:** 2022-11-22

**Authors:** Surabhi Naik, Akram Mohammed

**Affiliations:** ^1^ Department of Surgery, James D. Eason Transplant Institute, College of Medicine, University of Tennessee Health Science Center, Memphis, TN, United States; ^2^ Center for Biomedical Informatics, College of Medicine, University of Tennessee Health Science Center, Memphis, TN, United States

**Keywords:** host-pathogen interaction, correlation network, RNA-sequencing, immune response, candida albican, WGCNA, transplantation

## Abstract

Invasive fungal infections are a significant reason for morbidity and mortality among organ transplant recipients. Therefore, it is critical to investigate the host and candida niches to understand the epidemiology of fungal infections in transplantation. *Candida albicans* is an opportunistic fungal pathogen that causes fatal invasive mucosal infections, particularly in solid organ transplant patients. Therefore, identifying and characterizing these genes would play a vital role in understanding the complex regulation of host-pathogen interactions. Using 32 RNA-sequencing samples of human cells infected with *C. albicans*, we developed WGCNA coexpression networks and performed DESeq2 differential gene expression analysis to identify the genes that positively correlate with human candida infection. Using hierarchical clustering, we identified 5 distinct modules. We studied the inter- and intramodular gene network properties in the context of sample status traits and identified the highly enriched genes in the correlated modules. We identified 52 genes that were common in the most significant WGCNA turquoise module and differentially expressed genes in human endothelial cells (HUVEC) infection vs. control samples. As a validation step, we identified the differentially expressed genes from the independent Candida-infected human oral keratinocytes (OKF6) samples and validated 30 of the 52 common genes. We then performed the functional enrichment analysis using KEGG and GO. Finally, we performed protein-protein interaction (PPI) analysis using STRING and CytoHubba from 30 validated genes. We identified 8 hub genes (*JUN, ATF3, VEGFA, SLC2A1, HK2, PTGS2, PFKFB3, and KLF6*) that were enriched in response to hypoxia, angiogenesis, vasculogenesis, hypoxia-induced signaling, cancer, diabetes, and transplant-related disease pathways. The discovery of genes and functional pathways related to the immune system and gene coexpression and differential gene expression analyses may serve as novel diagnostic markers and potential therapeutic targets.

## Introduction

Solid organ transplant (SOT) patients are exposed to various complications, e.g., invasive fungal infection and organ failure, which are the major challenge in SOT and affect the morbidity and mortality in transplant patients. The most prevalent invasive fungal infection in SOT is Candidiasis, which includes about 60% of infections, followed by aspergillosis accounts for up to 25% of fungal infections (Shoham and Marr, 2012).

An opportunistic fungal pathogen, *Candida albican*s, is part of healthy human gut microbiota. However, when immunity is compromised or suppressed, particularly in organ transplant individuals, AIDS patients, chemotherapy-treated patients, and neonates, the mucosal layer becomes more susceptible to fatal invasive *C. albicans* infections such as candidiasis (Sangeorzan et al., 1994; Rhodus et al., 1997; Revankar et al., 1998; Redding et al., 1999; Willis et al., 1999), (Sobel, 1985). *C. albicans* can switch from an avirulent commensal yeast form to a virulent invasive hyphal form in which hyphae invade through the mucosal layer and disseminate/propagate through the blood, infecting other organs as well as developing multidrug resistance (Klepser, 2006; Cowen et al., 2015; Arendrup and Patterson, 2017; Pendleton et al., 2017; Nishimoto et al., 2020). In the process of *C. albicans* infection, the first site of host-pathogen interactions is epithelial and endothelial cells (Barker et al., 2008; Liu et al., 2015a). The development of invasive fungal diseases relies on the synergy between the host immune response and fungal virulence. Comprehensive network analysis is vital to understanding the regulatory network and rewiring to respond to these infections.

Recent efforts have been made for the functional and molecular characterization of *C. albicans* genes using RNA sequencing (Wu et al., 2016; Brown et al., 2019; Romo et al., 2019; Zhang et al., 2019; de Vries et al., 2020; Thomas et al., 2020; Xu et al., 2020). Numerous studies suggest gene biomarkers as potential therapeutic targets and diagnostic markers in various fungal infections (Dix et al., 2015; Huppler et al., 2017; Díez et al., 2021; Hamam et al., 2021). Weighted gene correlation network analysis (WGCNA) has been widely used in disease diagnosis (Liu et al., 2017; Liang et al., 2018; Tang et al., 2018; Li et al., 2019; Yin et al., 2019), physiology (Kadarmideen et al., 2011; Zuo et al., 2018; Chen et al., 2019), drug targets (Puniya et al., 2013; Maertens et al., 2018), and cross-species (Mueller et al., 2017) but has never been applied in the context of candida pathogenesis (Thomas et al., 2020). Therefore, we developed a novel approach to identify host-pathogen interactions in *C. albicans* and humans.

In this work, we applied WGCNA to analyze 32 RNA-seq samples from *in vitro* infection of *C. albicans* on human endothelial and oral epithelial cells after 1.5, 5, and 8-h of infection and controls. We identified 5 modules in human endothelial cells (HUVEC) human cell lines in infection vs. control status and separately identified differentially expressed genes (DEG). We reported the common genes across the two methods (WGCNA and DEG). We then validated a subset of genes using differential gene expression analysis of candida-infected human cell lines OKF6. Finally, we performed protein-protein interaction network analysis and identified hub genes that could be novel targets to investigate *C. albicans* infection in humans. Through these central genes’ biological and molecular functions, we gained insights into the signaling pathways previously not correlated with the fungal pathogen-host response and other diseases.

## Materials and methods

### Data collection

**FIGURE 1:**
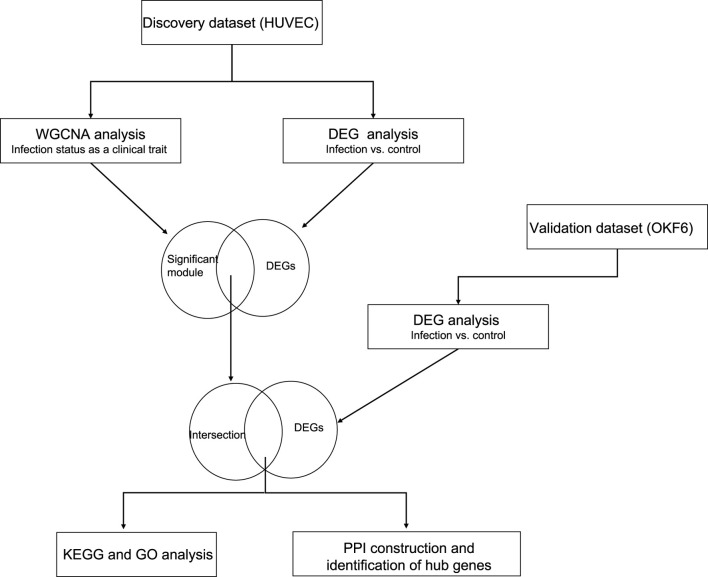
The schematic representation of the overall methodology: The discovery dataset was analyzed using two independent methods (WGCNA and DESeq2). Their intersecting genes were overlapped with the DEG list from the independent validation set to build the PPI network and identify the hub genes.

All processed gene expression datasets were collected from publicly available NCBI Gene Expression Omnibus GSE56093 (Liu et al., 2015a). The raw sequence data was aligned to the human and candida reference genomes separately by Liu et al. (Liu et al., 2015a), and the resultant count matrices were utilized for the WGCNA and DEG analyses. This dataset was comprised of 88 samples from *in vitro* and *in vivo* experiments. Of those, we only utilized 32 *in vitro* samples of human cell lines (endothelial and epithelial) infected with *C. albicans* (SC5314 and WO1 strains) and their controls at three different time points. More information is given in Supplementary Table S1. The overall methodology steps are shown in Figure 1.

### Data normalization and transformation

We performed normalization on the RPKM (reads per kilobase of transcript, per million mapped reads) values using the GCRMA limma package (Gautier et al., 2004) by first removing features with counts <10 in 90% of the samples, as these could be a potential cause of the noise. Then, we performed and compared three data transformation techniques, logarithmic, regularized logarithmic, and variance stabilizing transformation (Lin et al., 2008)^,^ to stabilize the variance across sample mean values. We chose the regularized log transformation due to stability (Supplementary Figure S1).

### Weighted gene coexpression network framework

We constructed the weighted gene coexpression network using the R WGCNA package (Langfelder and Horvath, 2008). The normalized data were used as input for network construction and gene module detection. It uses correlation to find functional modules of the highly correlated gene networks. First, we evaluated the soft threshold power (β) to convert coexpression into weight with a scale-free topology index of 0.9. We chose soft threshold powers of 8 to calculate the correlations between the adjacent genes (Supplementary Figure S2). Pearson correlations between each gene pair were calculated. We then converted this adjacency matrix into a topological overlap matrix (TOM) to define gene clusters that show the amount of overlap in shared neighbors of the gene network. The dissimilarity measure was determined for hierarchical clustering and module detection. Modules of clusters of genes with high topological overlap were selected using a dynamic tree-cut algorithm. Several modules were identified, and the modules with similar expression levels were merged by calculating their eigengenes corresponding to their correlations. We further determined the association of these modules with the external traits. We identified the genes with high gene significance (GS) and module membership (MM) in the turquoise and blue modules in HUVEC data. Last, intramodular connectivity was analyzed in human modules using MTR>0.35 and *p*-value < 0.05. All the categorical variables were binarized for the analyses.

### Identification of differentially expressed genes

Differentially expressed genes were identified using DESeq2 R Bioconductor package (Love et al., 2014). We used raw counts that were fed to the DESeq2 since it corrects for library size. The variance stabilizing transformations (VST) function estimated the sample differences (Lin et al., 2008). The statistical significance for the differentially expressed genes was set to q-value < 0.05 and log2 fold change (log2FC) > 1.

### Functional enrichment analysis of genes

We performed Gene Ontology (GO) and Kyoto Encyclopedia of Genes and Genomes (KEGG) enrichment analyses to study the role of the genes and identify their biological functions and pathways. Gene Ontology analysis was performed to determine the biological process. We considered an adjusted *p*-value threshold of ≤ 0.05 and a minimum gene count of 3 for the KEGG pathways and GO functional terms. As the contribution of all the genes is not the same, we identified hub genes and further investigated their function.

### Statistical analysis and data visualization

The R programming language (Horgan, 2012) was used to normalize the RNA-seq data. We conducted Fisher’s exact tests to identify the statistically significant Gene Ontology terms and functional classes. Enrichment analysis based on a hypergeometric test was implemented, and Benjamini Hochberg multiple testing was used to correct the *p*-value. Data visualization to show differentially expressed genes between infected and uninfected groups for top selected genes was plotted using the complex Heatmap function in R. The data visualization was performed using the cluster profiler package in R (Yu et al., 2012).

### Protein-protein interaction network analysis

The validated genes are uploaded into the STRING database, and high confidence interaction score ≥ 0.7 was used to reduce false-positive interactions (Bozhilova et al., 2019). The resultant network output was loaded into Cytoscape. CytoHubba (Chin et al., 2014) was used with the Maximal Clique Centrality (MCC) algorithm to discover the hub genes in the PPI network (Li and Xu, 2019).

## Results

### Network construction and module identification

**FIGURE 2:**
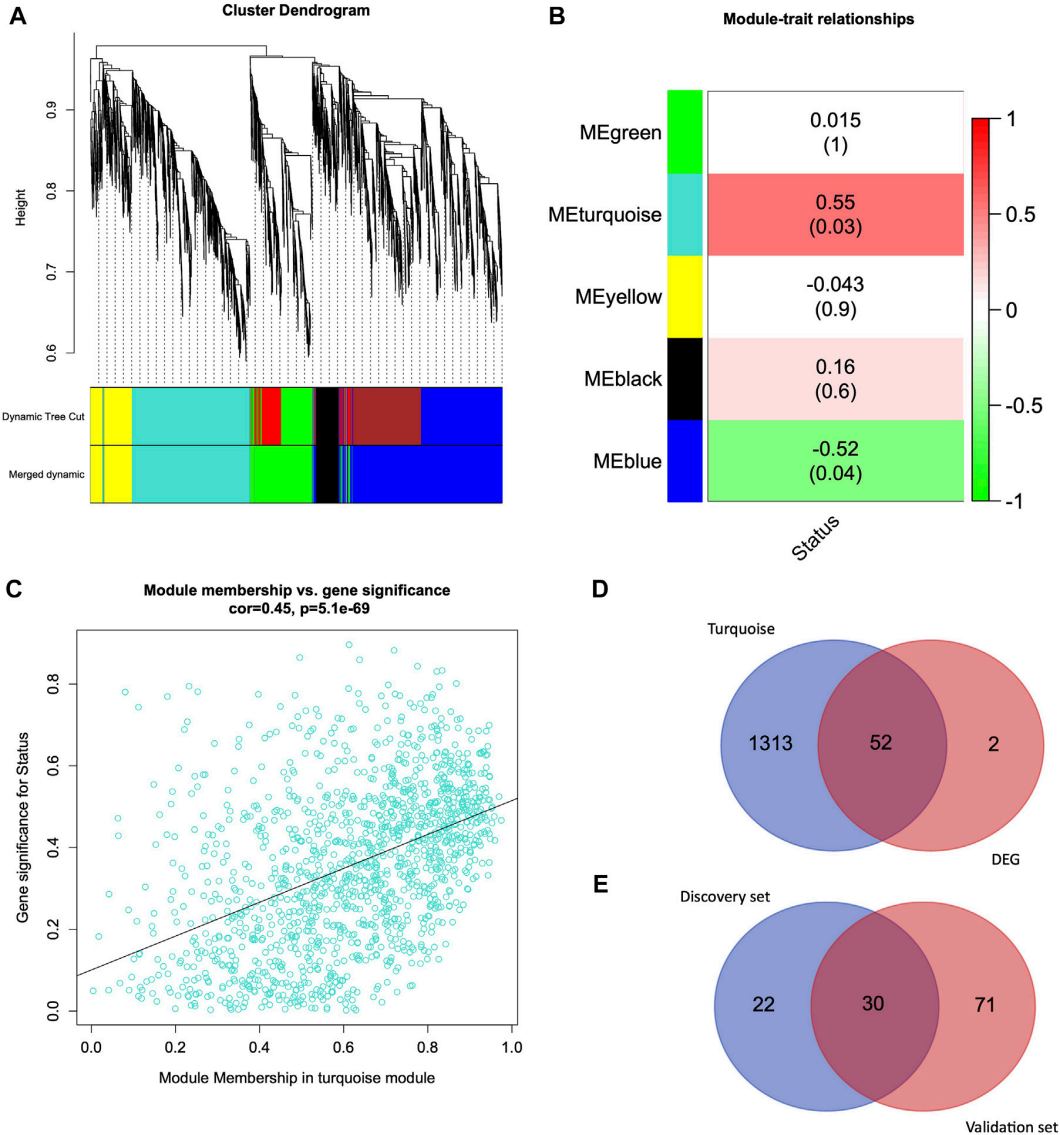
Weighted Gene Coexpression Network Analysis and Venn Diagram **(A)** Hierarchical clustering of 4,669 genes from HUVEC discovery dataset **(B)** Module-trait relationship exhibiting associations of module eigengenes with the clinical trait (infection status). **(C)** Relationship between turquoise module membership (MM) and gene significance (GS). **(D)** Venn diagram representing the overlapping genes from the turquoise module genes and differentially expressed genes. **(E)** Venn diagram representing the common genes between the discovery set genes (overlapping genes from WGCNA turquoise module and DEG genes) and differentially expressed genes from the validation set.

Weighted Gene Correlation Network Analysis was conducted on HUVEC data. We performed hierarchical clustering of genes using a topological overlap matrix and merged modules with similar expression profiles (Figure 2A). Each leaf corresponds to a gene, and branches correspond to the cluster of highly coexpressed genes. After cutting tree branches, we identified five different modules, turquoise, yellow, black, blue, and green, with 1,365, 459, 261, 1829, and 755 genes in HUVEC (Supplementary Table S2). A total of 4,669 genes were identified from the HUVEC data set, and in each module, the number of genes ranged between 261 and 1829.

### Module association with external traits

We further analyzed the module trait relationship (MTR) between the module eigengene and clinical traits, where each cell represents the correlation strength (red is positively correlated, and green is negatively correlated) with their corresponding *p*-value (Figure 2B). We demonstrate that some module eigengenes are highly correlated with infection (status traits). We observed that the turquoise (*r* = 0.55, *p* = 0.03) and blue (*r* = −0.52, *p* = 0.04) modules were highly correlated with the infection status in HUVEC cells. Since the turquoise module, with 1,365 genes, is the most significantly correlated with the clinical trait, we focused on this module for further analysis.

### Intramodular connectivity using gene significance and module membership

We quantified genes with high significance for the trait status of HUVEC and high module membership by comparing their similarities in every module. There was a highly significant correlation between gene significance and module membership in the turquoise module. Figure 2C represents the correlation between turquoise module membership and gene significance (*r* = 0.45, *p* = 5.1e-69).

### Differentially expressed genes and intersection with WGCNA

We used DESeq2 as a second independent method on the entire HUVEC dataset to identify 54 genes that were differentially expressed in the HUVEC (infection vs. control) samples (q-value < 0.05 and log2FC > 1). From the WGCNA analysis, we identified 1,365 genes in the most significantly correlated turquoise module (Figure 2B). When we further investigated the intersection of WGCNA and DEGs, 52 genes were common between the turquoise module and the DEG list (Figure 2D). The list of turquoise module genes, DEGs, and intersecting genes is given in Supplementary Table S4.

### Validation of candidate genes

In order to validate these 52 common genes, we utilized a validation dataset comprised of candida-infected human oral keratinocytes (OKF6 cell line). We performed the differential gene expression analysis on infection vs. control and identified 101 DEGs. When we overlapped these 101 genes with 52 common genes from the discovery dataset, we found 30 genes that were differentially expressed in the OKF6 validation dataset (Figure 2E and Supplementary Table S3). The following are the 30 validated genes: *SLC2A1, ATF3, JUN, KDM7A, DUSP1, PTGS2, NAB2, PIM1, MAFF, ADM, PFKFB3, KLF6, BNIP3, CSRNP1, VEGFA, ENO2, ANKRD37, PPP1R15A, KDM3A, ANGPTL4, BHLHE40, ARRDC3, SLC2A3, KLF7, DDIT4, ERRFI1, KLF4, FOSL2, EFNA1, and HK2.* The list of genes from the discovery set, validation set, and intersecting genes are given in Supplementary Table S4.

### Integrating network analysis with functional enrichment analyses

To understand the biological roles of these 30 validated genes, we performed GO and KEGG pathway analyses to identify the biological pathways that were significantly enriched (FDR ≤ 0.05) in these modules.

**FIGURE 3:**
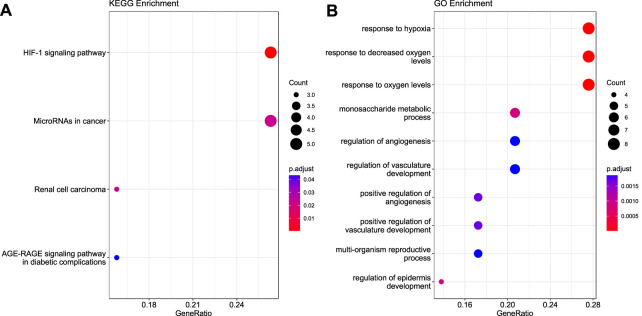
Functional enrichment analysis for the 30 validated genes. **(A)** KEGG pathway **(B)** Gene Ontology.

KEGG analyses revealed that the genes were highly enriched in the HIF-1 signaling pathway, microRNAs in cancer, renal cell carcinoma, and AGE-RAGE signaling pathway in diabetes complications, as shown in Figure 3A (Detailed information is provided in Supplementary Table S3). Gene Ontology analyses elucidated that these genes were enriched in response to hypoxia, monosaccharide metabolic process, angiogenesis regulation, vasculature development, reproductive process, and epidermis development, as shown in Figure 3B. Additional details are given in Supplementary Table S3.

### Protein-protein interaction network analysis

**FIGURE 4:**
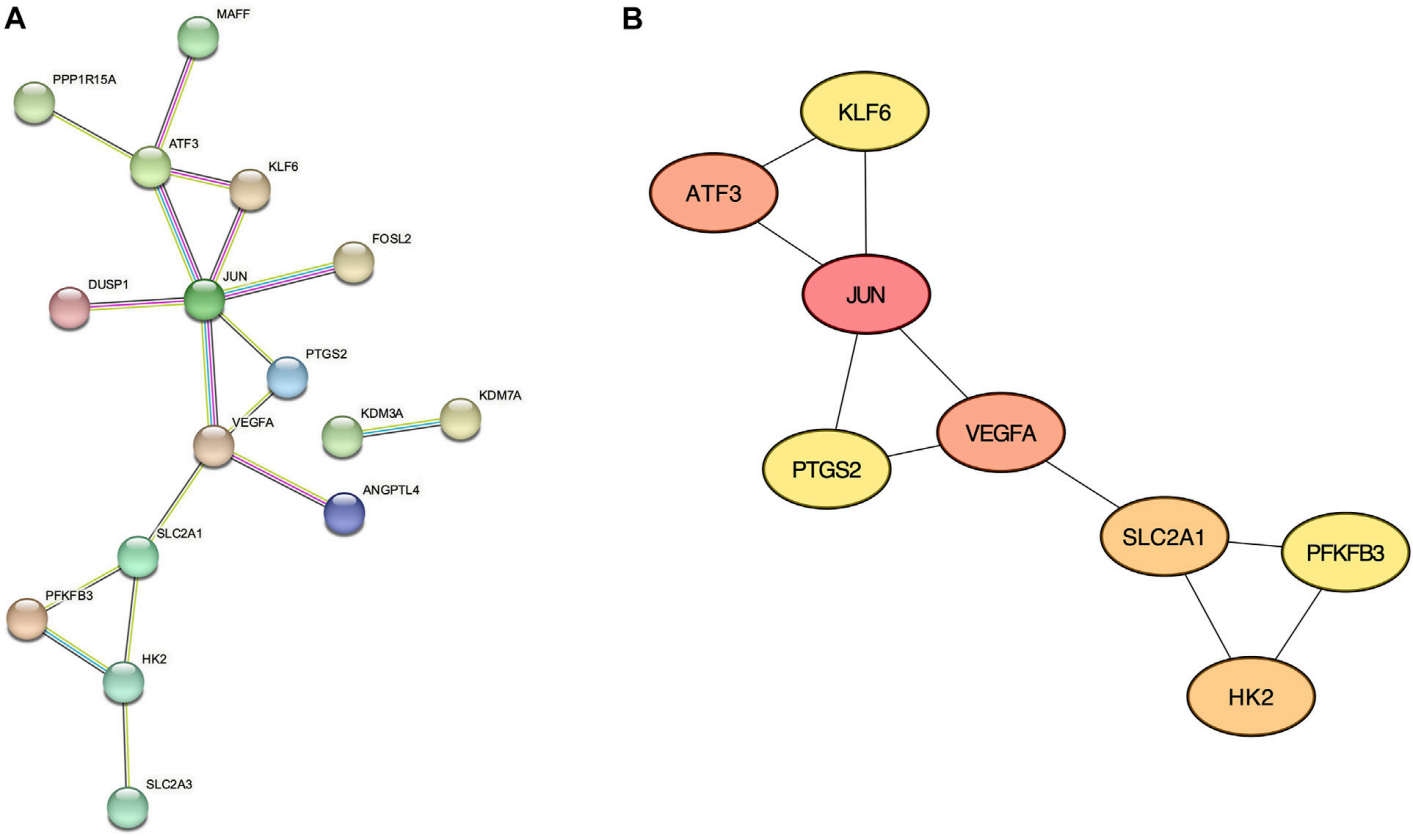
Protein-Protein interaction networks. **(A)** STRING analysis of validated genes. **(B)** CytoHubba with Maximal Clique Centrality analysis showing 8 hub genes.

From the 30 validated genes, we first performed the protein-protein interaction (PPI) analysis using the STRING database (Figure 4A). The resultant data is then imported to the Cytoscape plugin CytoHubba, and the top 8 genes with the highest Maximal Clique Centrality (MCC) score were considered hub genes: *JUN, ATF3, VEGFA, SLC2A1, HK2, PTGS2, PFKFB3, and KLF6* (Figure 4B and Supplementary Table S3).

## Discussion

The interaction between host cells and Candida is central to the immunopathology of candidiasis in transplant patients; a comprehensive understanding of this synergy will identify new treatment strategies. Here, we investigate how human epithelial and endothelial cells communicate with different Candida species during infection. In this study, we constructed a weighted gene correlation network and performed differential gene expression analysis to identify genes that are important in host‒candida interactions.

Comparative network analysis could rank genes for further investigation of their connectivity (Schadt et al., 2005). A distinct advantage of WGCNA is that it considers modules or gene clusters for constructing interactions, and the genes in a module are likely to be connected by the same regulatory pathways. Therefore, in this study, we aim to discover novel genes and molecular pathways in human-candida infection and to understand the regulation due to cell dynamics using the WGCNA and DESeq2 algorithms. Network depictions provided immediate insight into the relationships between the correlated modules. The construction of a gene coexpression network and differential gene expression analysis of the discovery and validation data set facilitated the identification of genes with similar biological functions by GO and KEGG analyses.

According to the results of functional enrichment analysis, the top 3 GO terms and topmost KEGG pathway were a response to hypoxia, response to decreased oxygen, response to oxygen levels (Figure 3B), and hypoxia-inducible factor 1 (HIF-1) signaling pathway (Figure 3A). HIF-1 is a transcription factor that functions as a master regulator of oxygen homeostasis. It has been shown that suppressing HIF-1 helps treat cancer and ischemia (Ziello et al., 2007). All organs during the process of transplantation undergo hypoxic and ischemic injury. Low oxygen levels trigger the colonization of candida infection in the human host, resulting in complications like allograft rejection in SOT patients (Akhtar et al., 2014). We identified eight hub genes using PPI network analysis. Four hub genes (*HK2, PFKFB3, SLC2A1, and VEGFA*) are involved in the HIF-1 signaling pathway. The hexokinase isoenzyme (*HK2*) elevates innate immunity in hepatocellular carcinoma (Perrin-Cocon et al., 2021). *HK2* and *PFKFB3* are involved in glycolysis which affects the immune response against fungal infection (Perrin-Cocon et al., 2021); specifically, after transplantation, the *PFKFB3* gene increase the risk of invasive pulmonary aspergillosis (Gonçalves et al., 2021). *Huang et al.* showed in their omics analysis that *SLC2A1* is involved in ischemic reperfusion injury in liver transplant patients and forms the core gene network (Huang et al., 2019a). Vascular Endothelial Growth Factor A (*VEGFA*) is associated with an increased risk of chronic kidney disease (Anderson et al., 2018) but induces vasculogenesis. Kidney vasculature comprises vascular smooth muscle and endothelial cells (Udan et al., 2012). One of the most challenging components to handle during a kidney transplant is through vasculogenesis and angiogenesis processes (Munro and Davies, 2018; Lebedenko and Banerjee, 2021). HIF-1 stimulates the *VEGFA* to maintain oxygen delivery and protect the kidney (Hunga et al., 2013).

The other top enriched KEGG pathways in our analysis were microRNAs in cancer (hsa05211) and renal cell carcinoma (hsa05206). MicroRNAs play a diverse role in cancer and infections (Yong and Dutta, 2009). Recent advances in microRNA therapeutics have shown the extensive use of microRNAs for cancer and infections (Rupaimoole and Slack, 2017). There has been increased support for microRNA therapeutics in solid organ transplantation, including kidney (Wilflingseder et al., 2015; Jin et al., 2017; Ledeganck et al., 2019), lung (Benazzo et al., 2022), and heart transplantation (Hamdorf et al., 2017). *Candida albicans* have been linked to cancerous processes by taking advantage of the compromised immune system (Ramirez-Garcia et al., 2016; Chung et al., 2017; Sultan et al., 2022). Our PPI network analysis identified four hub genes (*SLC2A1, VEGFA*, *JUN*, and *PTGS2*) enriched in the cancer-related pathways. *SLC2A1* belongs to a glucose transporter family and has been reported to be associated with HCC (Kim et al., 2017). *SLC2A1* is also essential to IRI during liver transplantation (Huang et al., 2019b) and a diagnostic biomarker for colorectal cancer (CRC) (Liu et al., 2022). In CRC, the *SLC2A1* gene infiltrates the CD4^+^ T cell, neutrophil, dendritic cells, and B cells (Liu et al., 2022). Candidiasis is one of the risk factors for Oral squamous cell carcinoma (OSCC). The transcriptomics data analysis revealed that *VEGFA* and *JUN* are highly regulated in OSCC invasion and metastasis (Vadovics et al., 2022). *JUN* is a member of the activator protein-1 family of oncogenic transcription factors, which is involved in various cancer-related and cell signaling pathways such as tumorigenesis, cell differentiation, and angiogenesis (Brennan et al., 2020). Post renal transplantation, the activation of c-JUN affects acute humoral rejection and acute T-cell-mediated rejection (Kobayashi et al., 2010). c-JUN is also associated with reduced graft function and plays an important role in renal pathophysiological events (Kobayashi et al., 2010). Prostaglandin E2 (PGE2) is an inflammatory mediator produced by the Prostaglandin-endoperoxide synthase (*PTGS2*) enzyme, and PGE2 promotes candida morphogenesis. In response to candida infection, PTGS2 activation promotes NF-kB and MAPK signaling pathways (Deva et al., 2003). In OSCC, *PTGS2* involves an inflammatory response to infection by promoting tumorigenesis (Cacina et al., 2018) and activating transcription factor 3 (*ATF3*), one of the 8 hub genes that regulate the *PTGS2* during acute inflammation (Hellmann et al., 2015) and helps in the homeostasis of the metabolism and immune system (Sha et al., 2017). Zhu et al. also showed that *ATF3* is one of the top hub genes in samples infected with 4 different candida species (Zhu et al., 2022). Using bioinformatics omics analysis, *ATF3* and Kruppel‐like factor 6 (*KLF6,* hub gene) are shown to be the central players in ischemic reperfusion injury in liver transplant patients (Huang et al., 2019b). *KLF6* promotes inflammation and oxidative stress by regulating HIF-1 expression in macrophages (Kim et al., 2020).

Another enriched KEGG pathway was the AGE-RAGE signaling in diabetes complications (hsa04933). Endoplasmic reticulum stress due to AGE-RAGE plays an essential role in renal inflammation, diabetic nephropathy (Pathomthongtaweechai and Chutipongtanate, 2020) and early-stage renal disease (Meerwaldt et al., 2009). Advanced glycation end products (AGEs) may also play a role in the hardening of arteries after renal transplantation (Liu et al., 2015b). Our two hub genes, *JUN* and *VEGFA,* showed enrichment in the AGE-RAGE signaling pathway in diabetes complications. Poorly controlled diabetes increases the risk of fungal infections (Rodrigues et al., 2019). Some diabetes-related complications include cardiovascular disease, kidney disease, neuropathy, hearing loss, vision loss, Alzheimer’s, liver disease, etc. (Deshpande et al., 2008; Prasad et al., 2016). *VEGFA* and *JUN* were identified as the central players in diabetic nephropathy (Oltean et al., 2015; Wang et al., 2021) and Alzheimer’s disease (Zu et al., 2021) whereas, *VEGFA* was associated with diabetic retinopathy (Bucolo et al., 2021), cardiac autonomic neuropathy (Ravichandran et al., 2019), and non-alcoholic fatty liver disease-hepatocellular carcinoma (Shen et al., 2022). Each hub gene plays a vital and diverse role in the pathways and biological processes. Therefore, more research is warranted on the divergent roles of these genes’ signaling and regulatory mechanisms during infection, cancer, and transplantation.

## Limitations

WGCNA lacks resolution as it decomposes a group of genes into a single eigenvalue that may not correctly represent a single gene’s expression profile or pathway changes. Further analysis may be needed to detect changes in the expression of individual processes. Another limitation of the study is the small sample size; therefore, we present this study as a proof of concept to be validated in a larger cohort. The current study used cell lines from epithelial and endothelial cells; thus, the identified gene markers should be validated from the peripheral blood transcriptome of candidiasis patients for non-invasive clinical relevance.

## Data Availability

Publicly available datasets were analyzed in this study. This data can be found here: https://www.ncbi.nlm.nih.gov/geo/query/acc.cgi?acc=GSE56093.
