# Supplementary figures and images for: Coexpression network analysis of human candida infection reveals key modules and hub genes responsible for host-pathogen interactions
Source: Front Genet. 2022 Nov 22;13:917636. doi: 10.3389/fgene.2022.917636 (PMC9722774; doi:10.3389/fgene.2022.917636)

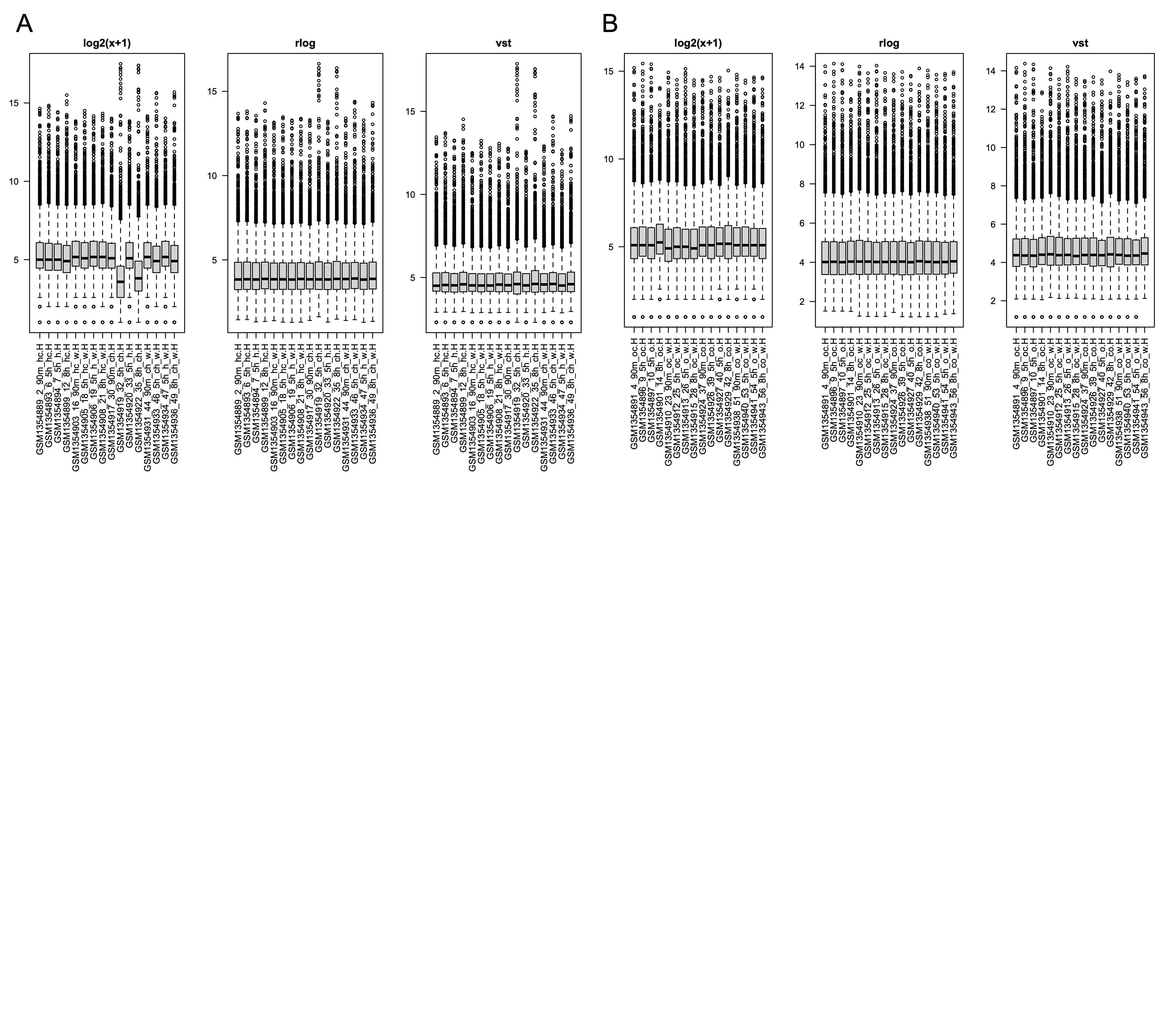

Supplement: Supplementary file 2 [file Image3.JPEG]

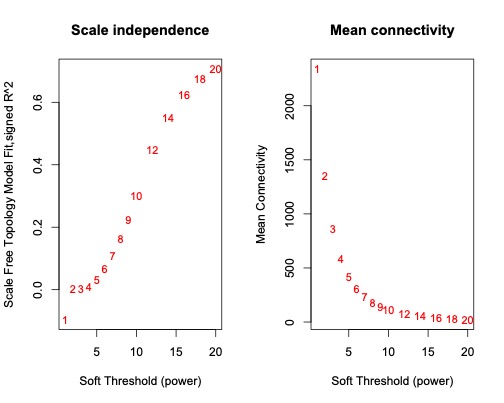

Supplement: Supplementary file 5 [file Image4.JPEG]
